# Murine roseolovirus does not accelerate amyloid-β pathology and human roseoloviruses are not over-represented in Alzheimer disease brains

**DOI:** 10.1186/s13024-021-00514-8

**Published:** 2022-01-15

**Authors:** Tarin M. Bigley, Monica Xiong, Muhammad Ali, Yun Chen, Chao Wang, Javier Remolina Serrano, Abdallah Eteleeb, Oscar Harari, Liping Yang, Swapneel J. Patel, Carlos Cruchaga, Wayne M. Yokoyama, David M. Holtzman

**Affiliations:** 1 Division of Rheumatology, Department of Pediatrics, Washington University School of Medicine, St. Louis, MO 63110 USA; 2 Department of Neurology, Hope Center for Neurological Disorders, Knight Alzheimer’s Disease Research Center, Washington University School of Medicine, St. Louis, MO 63110 USA; 3 Division of Biology and Biomedical Sciences (DBBS), Washington University School of Medicine, St. Louis, MO 63110 USA; 4 Present address: Genentech, 1 DNA Way, South San Francisco, CA 94080 USA; 5 Department of Pathology & Immunology, Washington University School of Medicine, St. Louis, MO 63110 USA; 6 Department Psychiatry, Washington University School of Medicine (WUSM), 660 S. Euclid Ave. B8134, St. Louis, MO 63110 USA; 7 NeuroGenomics and Informatics, Washington University School of Medicine, St. Louis, MO USA; 8 Division of Rheumatology, Department of Medicine, Washington University School of Medicine, St. Louis, MO 63110 USA

**Keywords:** Alzheimer’s disease, Human roseolovirus, Murine roseolovirus, Amyloid-beta, Neuroinflammation

## Abstract

**Background:**

The role of viral infection in Alzheimer Disease (AD) pathogenesis is an area of great interest in recent years. Several studies have suggested an association between the human roseoloviruses, HHV-6 and HHV-7, and AD. Amyloid-β (Aβ) plaques are a hallmark neuropathological finding of AD and were recently proposed to have an antimicrobial function in response to infection. Identifying a causative and mechanistic role of human roseoloviruses in AD has been confounded by limitations in performing in vivo studies. Recent -omics based approaches have demonstrated conflicting associations between human roseoloviruses and AD. Murine roseolovirus (MRV) is a natural murine pathogen that is highly-related to the human roseoloviruses, providing an opportunity to perform well-controlled studies of the impact of roseolovirus on Aβ deposition.

**Methods:**

We utilized the 5XFAD mouse model to test whether MRV induces Aβ deposition in vivo. We also evaluated viral load and neuropathogenesis of MRV infection. To evaluate Aβ interaction with MRV, we performed electron microscopy. RNA-sequencing of a cohort of AD brains compared to control was used to investigate the association between human roseolovirus and AD.

**Results:**

We found that 5XFAD mice were susceptible to MRV infection and developed neuroinflammation. Moreover, we demonstrated that Aβ interacts with viral particles in vitro and, subsequent to this interaction, can disrupt infection. Despite this, neither peripheral nor brain infection with MRV increased or accelerated Aβ plaque formation. Moreover, −omics based approaches have demonstrated conflicting associations between human roseoloviruses and AD. Our RNA-sequencing analysis of a cohort of AD brains compared to controls did not show an association between roseolovirus infection and AD.

**Conclusion:**

Although MRV does infect the brain and cause transient neuroinflammation, our data do not support a role for murine or human roseoloviruses in the development of Aβ plaque formation and AD.

**Supplementary Information:**

The online version contains supplementary material available at 10.1186/s13024-021-00514-8.

## Background

In the past decades, major strides have been made to uncover the heterogeneous factors that contribute to the etiology of Alzheimer Disease (AD). Recent advancements in biomarker studies predict that amyloid-β (Aβ) plaques begin to aggregate in the brains of AD patients 15–20 years before the onset of cognitive symptoms. Aβ accumulation triggers a local inflammatory response in the central nervous system (CNS) and promotes subsequent intracellular tau accumulation in the neocortex [1]. However, the initial events that drive Aβ seeding remain under debate and recently, pathogens have been raised as contributing factors. Although the mechanistic contributions of pathogens are unclear, multiple pathogens have been associated with AD. Recent studies have suggested that Aβ may act as an antimicrobial peptide that binds to pathogens and limits their entry [2–6]. In these studies, Aβ seeding was proposed to accelerate as part of an immune response to infection, and repeated or chronic infection results in persistent inflammation and Aβ accumulation.

Herpesviruses have been considered in the pathogenesis of AD because they are ubiquitous in the population, cause chronic infections that periodically reactivate, and several herpesviruses, including herpes simplex viruses (HSV-1 and HSV-2), cytomegalovirus, Epstein-Barr virus, and the human roseoloviruses infect the CNS and cause neuroinflammation [7, 8]. Indeed, the herpesviruses have been associated with AD [9–14]. The human roseoloviruses, HHV-6A, HHV-6B and HHV-7, have received particular attention given their link with CNS diseases, such as multiple sclerosis and encephalitis [15, 16]. Moreover, there are reports of increased HHV-6 DNA in AD brains compared to non-AD controls [17], decreased HHV-6 IgG titers in the blood of AD patients compared to controls [6], and a human leukocyte antigen subtype associated with increased susceptibility to HHV-6A infection that was suggested to be an AD genetic risk factor [18]. A recent publication by Readhead, et al. utilizing transcriptomic, genomic and proteomic analysis of several brain banks suggested a link between human roseoloviruses, specifically HHV-6A and HHV-7, and AD [19]. Furthermore, another study observed Aβ aggregating around HHV-6 in vitro [5]. However, a reanalysis of the study published by Readhead, et al. using a different statistical methodology [20], as well as an additional cohort study of brain banks using transcriptomics and digital droplet PCR [14], both suggested a lack of association between human roseoloviruses and AD.

Establishing a causal link between human roseoloviruses and AD is hindered by the high prevalence and chronicity of infection [21, 22]. The beta-herpesviruses demonstrate species-specific tropism, therefore in vivo studies of human roseoloviruses have been limited to immunodeficient humanized mice or transgenic mice expressing the receptor for HHV-6A, CD46 [23–25]. While these are useful models to study human roseoloviruses, there are clear restraints. The humanized mouse models are immunodeficient and infection is limited to transferred human cells, whereas the CD46 transgenic mouse models utilize intracranial infection and are limited to HHV-6A infection [23–25]. On the other hand, murine roseolovirus (MRV) is genetically and morphologically highly-related to the human roseoloviruses and likely has high prevalence in wild mouse populations [26–28]. Neonatal infection with MRV results in transient failure to gain weight, thymic atrophy and CD4^+^ T cell depletion [26, 29]. We have previously shown that viral DNA was observed in the CNS after neonatal infection, although viral replication was not evaluated [26]. Furthermore, MRV DNA appeared to persist at low levels into adulthood, suggesting it establishes life-long latency similar to other herpesviruses.

Given the similarities of MRV to human roseoloviruses and its ability to infect the CNS, herein we investigated the impact of MRV on Aβ accumulation in vivo using the 5XFAD mouse model that overexpresses human *APP* and *PSEN1* transgenes with five AD-linked mutations [30]. We demonstrated that 5XFAD mice are susceptible to MRV infection and observed expression of late viral transcripts in the brain during acute infection, suggesting active viral replication. Additionally, we detected inflammation in the brain after peripheral and direct central nervous system (CNS) infection. Furthermore, Aβ interacts with MRV particles in vitro and, subsequent to this interaction, can disrupt infection. Despite the presence of MRV in the brains of 5XFAD mice, we did not detect increased accumulation of Aβ aggregates. Moreover, when we used RNA-sequencing (RNAseq) to evaluate a cohort of brains from Knight-ADRC research participants who either were cognitively unimpaired without AD or who had dementia due to AD at time of death, we found there was no association between HHV-6 infection frequency and AD. Taken together, our data demonstrate that although MRV infects the brain and induces both an acute peripheral and central inflammatory response, MRV does not increase Aβ aggregation in the brains of 5XFAD mice. Similarly, we found no association of HHV-6 RNA prevalence in AD human brains.

## Methods

### Animals

5XFAD (B6SJL-Tg (APPSwFlLon,PSEN1*M146L*L286V)6799Vas/Mmjax) and B6SJLF1/J mice were purchased from Jackson Laboratories. BALB/c mice were purchased from Charles River Laboratories. Mice were bred in house under pathogen-free conditions. Mice that were MRV-infected were housed in separate cages from mock-infected mice to avoid horizontal transmission. For all neonatal infections, male and female mice were infected and included in the experiments (sex ratio overall was ~ 50%). For infection of adult mice, female mice were used for all studies. These studies were conducted in accordance with institutional ethics guidelines in place through institutional animal care and use committee (IACUC) protocols approved by the Animal Studies Committee of Washington University in St. Louis.

### Virus stock and infection

MRV stocks were prepared from in vivo passaging in BALB/c mice as described previously [26]. For peripheral infection, mice received intraperitoneal (i.p.) inoculation using a 30-guage needle with 50 μL of a 1:5 dilution of viral stock in serum-free DMEM for a dose of ~ 2 × 10^7^ MRV genome copies. Mock infection was performed similarly with 50 μL of serum-free DMEM.

For intracranial intrahippocampal injections, virus stock was semi-purified through a sorbitol cushion as described previously [31, 32], with several modifications. Briefly, MRV virus stocks were prepared from minced thymus. Cells were pelleted by centrifugation, then were lysed using a cup horn sonicator. Large cellular debris was pelleted by centrifugation and supernatant was removed and layered onto a 20% sorbitol cushion (20% d-sorbitol, 50 mM Tris-HCl, pH 7.2, 1 mM MgCl_2_) at 55,000×g for 1 h using an ultracentrifuge. The remaining pellet was resuspended in 1% fetal bovine serum (FBS) in DMEM or PBS. For intracranial infection with semi-purified MRV stocks in 1% (FBS), six-week-old 5XFAD received bilateral, intrahippocampal injections of MRV with a 5 μL Hamilton syringe with a 30-gauge needle attached to a Kopf stereotaxic instrument (4 × 10^6^ MRV genome copies; 2 μL/site; AP: -2.0, ML: + 1.6/− 1.6, DV: − 2.0; 0.15 μL/min infusion rate). The needle was left undisturbed for 5 additional minutes after the injection before withdrawal. All mice were sacrificed 72 h post infection. To evaluate the impact of Aβ on infection, semi-purified viral stock in PBS was incubated with DMSO control or oligomeric-Aβ_42_ at a concentration of 10 μM for 2 h, then diluted 1:5 in PBS and 50 μL was injected intraperitoneally into postnatal day 0 (P0) BALB/c mice.

### Nanogold-labeled Aβ sample preparation

Human Aβ_42_ peptides (Cat#: 107761–42-2, GenScript) were resuspended with 1 mL of 1,1,1,3,3,3-Hexafluoro-2-propanol (HFIP). HFIP was then evaporated in a chemical hood overnight to obtain dry monomeric Aβ_42_ layer that was stored at − 20 °C in aliquots. On day of use, one 100 μg aliquot was dissolved with 5 μL of DMSO and diluted with sodium bicarbonate buffer (pH = 8.0) to make 10 μM monomeric Aβ_42_. Next, 10 nmol of Mono-Sulfo-N-Hydroxy-Succinimido- Nanogold® Labeling Reagent (Cat#: 2025, Nanoprobes) was added to the solution and incubated at RT overnight. Excess nanogold molecules were removed via three-day dialysis in H_2_O with 3.5 kDa cut-off tube at 4 °C. After dialysis, dry nanogold-labeled or unlabeled Aβ_42_ was obtained after overnight speed vacuum centrifugation at RT. The nanogold-labeled Aβ_42_ was dissolved with 5 μL of DMSO and used for preparation of monomeric, oligomeric Aβ_42_ and Aβ_42_ fibrils following a published protocol [33]. Briefly, oligomeric Aβ_42_ was formed using F12 media at 4 °C and Aβ_42_ fibrils were formed in 10 mM HCl at 37 °C for at least 24 h.

### Negative staining transmission electron microscope (NS-TEM) imaging

Before NS sample preparation, 10 μM of monomeric, oligomeric and fibrillar nanogold-labeled Aβ_42_ was mixed with purified MRV samples and incubated for 2 h. Afterwards, all NS samples were vortexed and prepared following standard uranyl formate negative staining protocol. At least 50 TEM images containing viral particles per sample were obtained with JEOL JEM-1400 120 kV TEM with 80,000x magnitude. The quantification of nanogold-labeled Aβ_42_ interaction with MRV particles was conducted by a blinded reviewer scoring for nanogold negative or positive viral particles.

### Tissue harvesting

#### Perfusion

Mice were anesthetized with Fatal-Plus (pentobarbital, 200 mg/kg, intraperitoneal) and transcardially perfused for three minutes with chilled phosphate-buffered saline (PBS) solution containing 0.3% heparin.

#### Brain extraction

Brains were hemisected: one hemisphere was dissected for specific brain regions, flash-frozen on dry ice, and stored at − 80 °C for biochemical and gene transcript analyses; the other hemisphere was fixed in 4% paraformaldehyde for 24–48 h and cryoprotected in 30% sucrose at 4 °C before sectioning for histology.

#### Spleen and thymus collection

Spleen and thymus dissection was performed as described previously [29]. Briefly, for nucleic acid analysis tissue was dissected and stored at − 80 °C or prepared for flow cytometry as described below.

### Histology and quantification

#### Sectioning

Hemibrains were sectioned coronally at 50 μm into six series using a freezing, sliding microtome (Leica). Brain slices were then stored at − 20 °C in cryoprotectant solution (0.2 M PBS, 15% sucrose, 33% ethylene glycol) until use. All histological studies were conducted by staining for two to three tissue slices from each mouse, separated by 300 μm (bregma − 1.5, − 1.8, and − 2.1 mm or − 1.8, and − 2.1 mm).

#### Immunostaining with DAB

Brain tissue staining was performed at room temperature and on a shaker as previously described [34]. Briefly, tissue sections were rinsed 3 times for 5 min each with Tris-buffered saline (TBS) and then blocked for nonspecific binding with 3% milk in TBS containing 0.25% Triton-X100 (TBS-X) for 30 min. Sections were then incubated at 4 °C overnight using primary antibodies (biotinylated anti-Aβ_1–13_ monoclonal antibody HJ3.4B (produced in-house, 2 μg/mL) for Aβ, mouse monoclonal anti-tau (gift from L. Binder, Northwestern University, 1:500) for Tau5, mouse monoclonal anti-phospho-tau Ser202, Thr205 (Thermo Fisher Scientific, 1:500) for AT8 phosphorylated tau, and rabbit anti-Iba1 (Wako, 019–19,741, 1:5000) for microglia with biotinylated goat anti-rabbit IgG secondary antibody (Thermo Fisher Scientific, 31,820, 1:1000). The following day, tissue sections were rinsed 3 times for 5 min each with TBS and incubated for 1 h in VectaStain Elite ABC-HRP Kit, Peroxidase (Vector Laboratories, PK-6100) and developed with DAB Eqv Peroxidase (HRP) Substrate (Vector Laboratories, SK-4103) following the manufacturer protocols. Glass slides were cover-slipped with cytoseal60 and allowed to air dry at room temperature for at least 24 h. Stained brain sections were scanned using a Nanozoomer 2.0-HT slide scanner (Hamamastu Photonics) at 20X magnification, courtesy of the Hope Center Alafi Neuroimaging Core.

#### Quantification

Images were quantified using Fiji software version 1.52v (ImageJ). The percent area covered by immunostaining was calculated by setting a single threshold per staining experiment to traced cortical and hippocampal regions. Two to three brain sections per mouse were averaged for an individual biological replica.

### Tissue lysate extraction and ELISA

#### Brain homogenization

Mouse posterior cortices were first homogenized in chilled PBS containing Complete Protease Inhibitor and phosSTOP Phosphatase Inhibitor (“Soluble fraction”), and then in 5 M guanidine-HCL buffer, pH 8.0 (“Insoluble fraction”) using magnetic beads (Next Advance, Bullet Blender Storm 24).

#### Elisa

ELISAs were performed using 96-well, half-well plates and concentrations were normalized to brain tissue weight. All antibodies were made in-house: anti-Aβ_35–40_ HJ2 (capture antibody for Aβ_40_), anti-Aβ_37–42_ HJ7.4 (capture antibody for Aβ_42_), biotinylated anti-Aβ_13–18_ HJ5.1B (detection antibody for Aβ_40_ and Aβ_42_). Aβ sandwich ELISAs were performed as previously described and concentrations were measured using Gen5 software version 1.11 with Synergy 2 (BioTek) [35] Samples were loaded in duplicates, averaged per mouse for an individual biological replicate.

### Nucleic acid preparation and analysis

DNA was extracted from organs using the QIAamp DNA Mini Kit (Qiagen) while RNA was extracted from organs using the RNeasy Plus Kit (Qiagen) per manufacturer protocols. qPCR of DNA was performed using TaqMan Universal Master Mix II (Applied Biosystems). RNA was treated with DNase prior to being analyzed by qPCR using TaqMan RNA-to C_T_ 1-Step Kit (Applied Biosystems). qPCR was analyzed on a StepOnePlus real time PCR machine (Applied Biosystems). For MRV *ORF69* standard curves were created using a plasmid of known base pairs in order to calculate copies/mL. For all other, relative expression compared to *Actb* (beta-actin) was calculated using ∆∆Ct. Primers used included: ORF69, Actb, IL-6, Iba1, ApoE, IL-1b, TREM2, GFAP.

### Flow cytometry

Spleen and thymus were prepared for flow cytometry by first crushing through a 70-μm cell strainer to obtain single cell suspensions. Red blood cells were lysed then cells were counted on a hemocytometer. Cells were stained with fixable viability dye (eBioscience) then incubated with 2.4G hybridoma supernatant to block Fc receptors. Surface staining was performed with the following fluorescent labeled antibodies: anti-CD3ε (145-2C11), anti-CD4 (RM4–5), anti-CD8α (53–6.7), and anti-CD44 (IM7) from Fisher Scientific, anti-CD19 (6D5), anti-NKp46 (29A1.4) and anti-CD62L (MEL-14) from Biolegend, and anti-CD45.2 (104) from eBioscience. Flow cytometry was performed on a FACS Canto (BD Biosciences) and analyzed using FlowJo v10 (TreeStar, Ashland, OR).

### RNA-seq data pre-processing

Human parietal RNA-seq was generated from the Charles F. and Joanne Knight Alzheimer’s Disease Research Center (Knight-ADRC) participants in two batches using Ribo-zero 150 × 2 pair-end reads. A detailed description of the sample selection criteria, extracted brain tissues, and the pipeline used for pre-processing and analyzing the human bulk RNA-seq data from this cohort is described elsewhere [36–39]. For this study, we used a subset of samples that are either definitive AD or controls. Precisely, the subset of samples retrieved from the Knight-ADRC consisted of 350 samples diagnosed with AD and 31 healthy controls. The obtained sequence files were in .bam format and already mapped to GENCODE [40] annotated human reference genome (GRCh38) using STAR software [41] in chimeric read detection mode. Unaligned reads from these aligned bam files were extracted using the “view -b -f 4” command supported in the samtools suite (http://samtools.sourceforge.net/). An unmapped BAM (uBAM) was created including these reads by using the “RevertSam” command supported in the picard tool suite (https://broadinstitute.github.io/picard/).

### Viral screening using RNA-seq data

The unaligned RNA-seq reads (uBAM) files were screened for a collection of microbes including HHV-6A and HHV-6B by using the “PathSeq” tool developed by the BROAD Institute (http://software.broadinstitute.org/pathseq/), using the pre-built microbe reference files (pathseq_microbe.fa.img). The total number of microbes screened for observing their abundance in the selected RNA-seq samples was 25,799 together with 118 human viruses. We used the “PathSeqPipelineSpark” command supported in the gatk tool suite (https://software.broadinstitute.org/gatk/download/) for running the “PathSeq” tool. The command was executed with default parameters (https://gatkforums.broadinstitute.org/gatk/discussion/10913/how-to-run-the-pathseq-pipeline) and required pre-built references were downloaded from the BROAD Institute (https://software.broadinstitute.org/gatk/download/bundle). The virus detection scores per individual sequence file output from the “PathSeq” tool were then imported into R (https://cran.r-proiect.org/), collated by cohort and virus types, visually inspected and plotted using “ggplot2” R package (version 3.3.3) [42].

### Statistical analysis of PathSeq results

Based on the number of samples that were positive and negative for HHV-6A and HHV-6B, two vectors were produced containing normalized viral abundance scores, and a non-parametric Wilcoxon Rank Sum test [43] was performed to determine if there was any relationship between viral abundance and AD classification. The Wilcoxon Rank Sum test implementation in the base R packages (version 3.5.2) [44] was used for this purpose.

### Statistical analysis

**Fig. 1:**
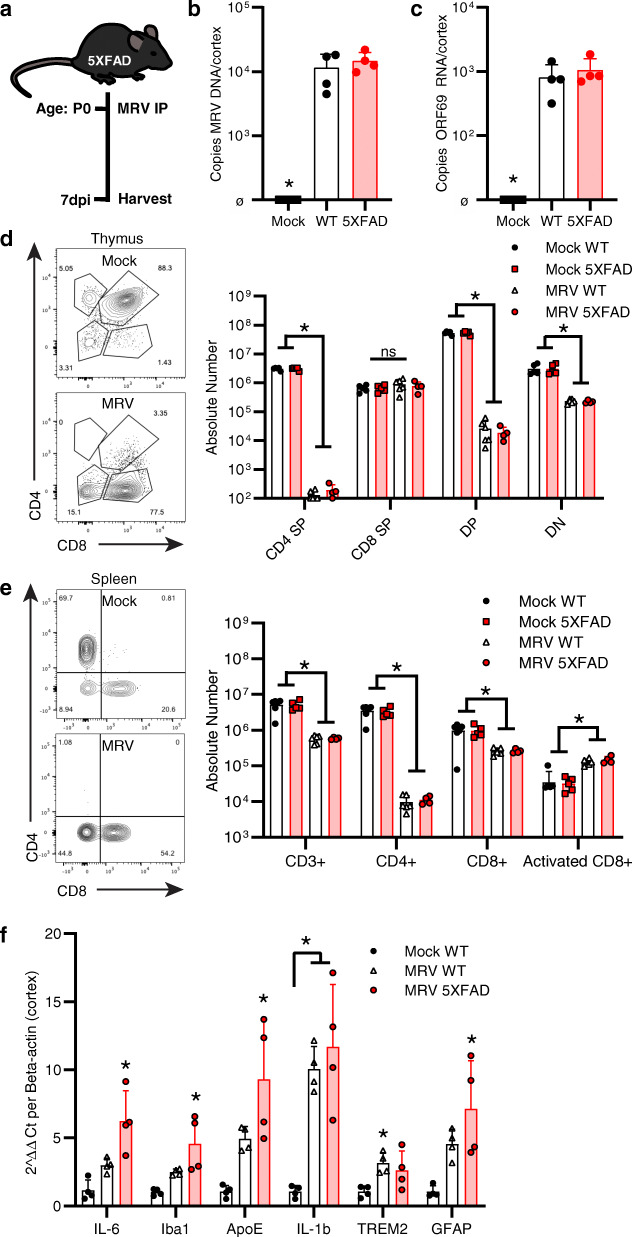
MRV infects the CNS and elicits a peripheral and central inflammatory response in 5XFAD mice. **A** Schematic of experimental paradigm. **B**, **C**, Copies/mL of MRV DNA **B** and ORF69 RNA **C** in cortex of 5XFAD mice infected with mock or MRV 7 dpi (i.p.). **D**, **E**,  Flow cytometry of absolute number of CD45^+^CD19^−^NKp46^−^ CD4^+^ or CD8^+^ thymocytes **D** and CD45^+^CD19^−^NKp46^−^CD3^+^ CD4^+^ or CD8^+^ T cells in the spleen **E**. **F** Inflammatory gene expression changes in cortical tissue. *P < 0.05 between WT mock and 5XFAD MRV. DP: CD4^+^CD8^+^ double positive. DN: CD4^−^CD8^−^ double negative. ø represents below levels of detection. Dpi: days post infection. Data expressed as mean ± SD, one-way ANOVA with Tukey’s post hoc test (two-sided) (b, c, d, e, f). *P* < 0.05. ns = not statistically significant. No other statistical comparisons are significant unless indicated

**Fig. 2:**
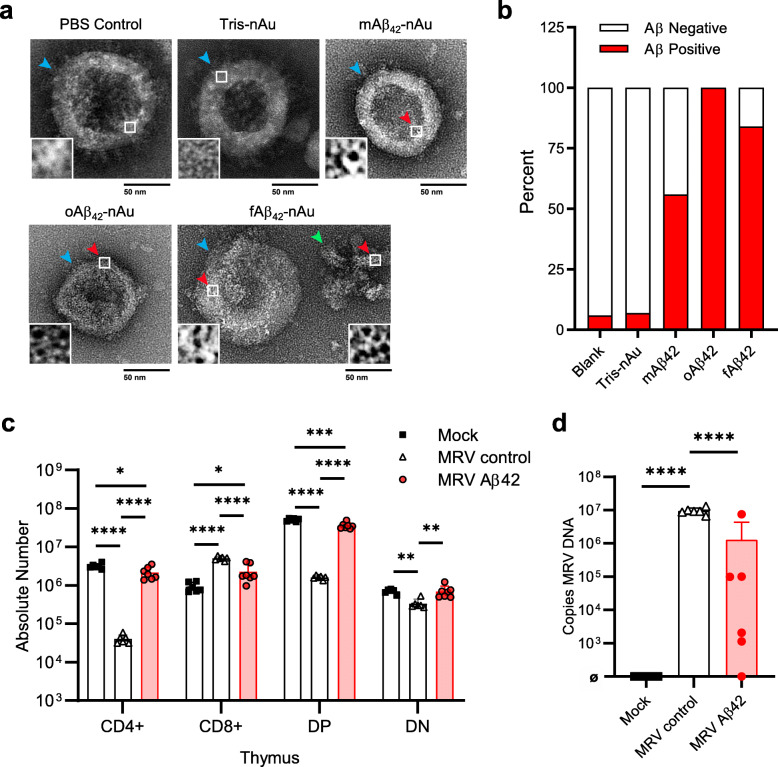
Aβ interacts with MRV in vitro and limits infection in vivo. **A** Interactions between purified MRV stock incubated for 2 h with PBS, Tris-nAu control, monomeric Aβ_42_ (mAβ_42_), oligomeric Aβ_42_ (oAβ_42_), or fibrillar Aβ_42_ (fAβ_42_) labeled with nanogold. Blue arrowhead: Viral particle. Red arrowhead: nanogold^+^ labeling. Green arrowhead: Fibrillar Aβ. A representative image with scale bar = 50 nm shown for each image with 5X increased magnification inset (white box represents area of inset) in the bottom left of image for viral particles or right of image for fibril. **B** Quantification of viral particles positive for nanogold (at least 50 particles were scored for each condition). **C**, **D**, Purified MRV stock incubated with PBS (MRV control) or oligomeric Aβ_42_ (MRV Aβ_42_) for 2 h, then i.p. injected into P0 BALB/c mice. Thymus was collected 7 dpi and evaluated for absolute number of CD4^+^, CD8^+^, DP (CD4^+^CD8^+^ double positive) or DN (CD4^−^CD8^−^ double negative) **C**, or MRV DNA load as copies/mL in the thymus **D**. ø represents below levels of detection. Data expressed as mean ± SD, one-way ANOVA with Tukey’s post hoc test (two-sided). *P < 0.05, ** *P* < 0.01, *** *P* < 0.001, and **** *P* < 0.0001. No other statistical comparisons are significant unless indicated

**Fig. 3:**
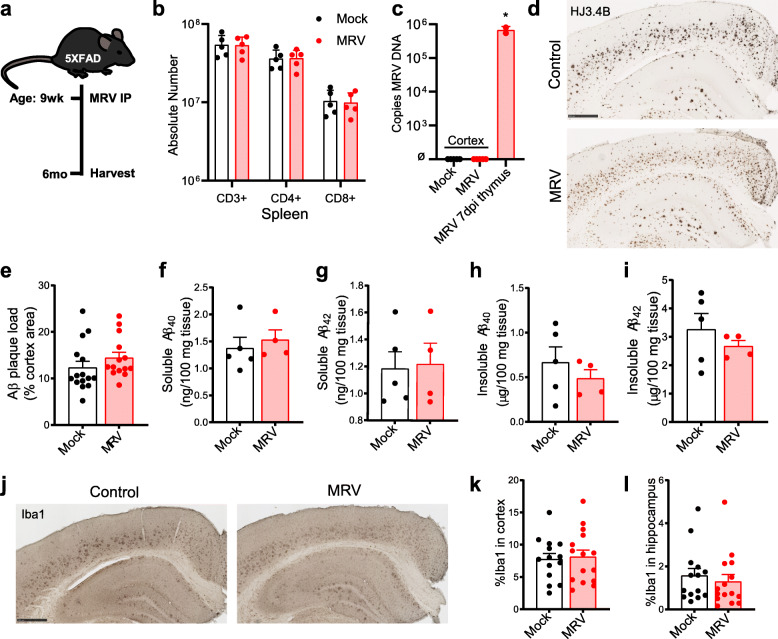
Peripheral MRV infection in adult 5XFAD mice does not affect Aβ plaque burden. **A** Schematic of experimental paradigm. **B** Absolute number of CD3^+^, CD4^+^, or CD8^+^ T cells in the spleen of 6-months-old 5XFAD mice infected with mock or MRV (i.p.). **C** Copies/mL of MRV DNA in cortex at 6-months-old or 7 dpi in thymus. **D**, **E**, Representative images of HJ3.4B staining for pan-Aβ immunoreactivity **D** and quantification of percent area coverage in the cortex **E**. Scale bar: 500 μm. **F–I**, Protein concentrations of PBS-soluble Aβ_40_ (f) and Aβ_42_ (g), or 5 M guanidine-soluble (“insoluble”) Aβ_40_ **H** and Aβ_42_ **I** in the cortex. **J–L**, Representative images of Iba1 staining for microglia **J** and quantification of percent area coverage in cortex **K** and hippocampus **L**. Scale bar: 500 μm. ø represents below levels of detection. Data expressed as mean ± SEM , ** –**, multiple t-tests using Holm-Sidak method **B**, one-way ANOVA with Tukey’s post hoc test (two-sided) **C**. *P < 0.05. No statistical comparisons are significant unless indicated

**Fig. 4:**
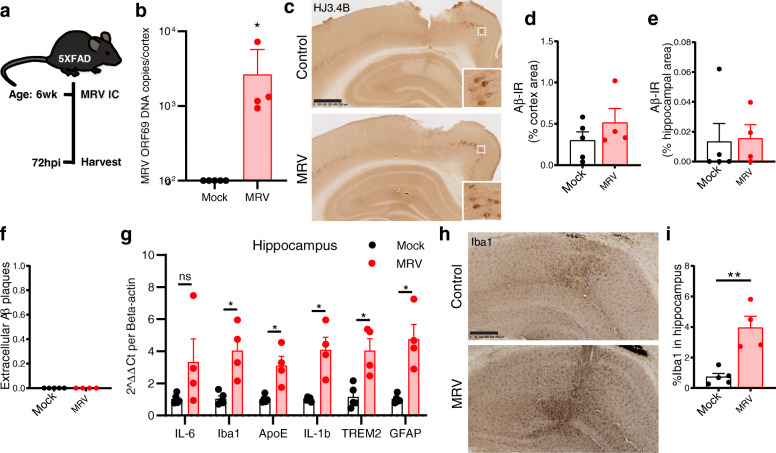
CNS MRV infection in adult 5XFAD does not accelerate Aβ formation but induces neuroinflammation. **A** Schematic of experimental paradigm. **B** Copies of MRV ORF69 DNA 72 hpi of mock or MRV, delivered bilaterally into the hippocampus of 6-week-old 5XFAD mice. **C–E**, Representative images of HJ3.4B staining for pan-Aβ immunoreactivity (Aβ-IR) with insets depicting intracellular Aβ-IR **C** and quantification of percent area coverage in the cortex **D** or hippocampus **E**. **F** Quantification of extracellular Aβ plaque deposits in cortex overlaying hippocampus. **G–H**, CNS inflammatory response to MRV in the hippocampus measured by gene transcript changes **G** and Iba1^+^ immunostaining for microglia (**H**, **I**). hpi: hours post injection. Scale bar: 500 μm. Data expressed as mean ± SEM, student’s t-test (b, d, e, f, h), and multiple t-tests **F**. *P < 0.05, ** *P* < 0.01. ns = not statistically significant. No statistical comparisons are significant unless indicated

Figures 1, 2, 3, 4, Supplementary Figs. 1, 2, 3: GraphPad Prism 8.0.2 was used to perform statistical analyses. Data are presented as means ± SD/SEM. No other statistical comparisons were significant unless otherwise indicated. *For two groups*: Statistical significance with normally distributed data was performed using a Student’s t-test (two-tailed). For data with unequal standard deviations, a t-test with Welch’s correction was performed. *For three or more groups*: Gaussian distribution of data was checked with the Anderson-Darling, D’Agostino and Pearson, and Shapiro-Wilk normality tests. One-way analysis of variance (ANOVA) followed by Tukey’s post hoc test was used to determine statistical significance. Asterisks represent *P* values as follows: * = *P* < 0.05, ** = *P* < 0.01, *** = *P* < 0.001, *** = *P* < 0.0001.

## Results

### 5XFAD mice are susceptible to MRV infection and develop neuroinflammation

We previously demonstrated that neonatal, peripheral MRV infection of BALB/c and C57BL/6 wild-type (WT) mice resulted in MRV DNA in the CNS, depletion of CD4 single positive (SP) and CD4CD8 double positive (DP) thymocytes, CD4^+^ T cells in the periphery, and peripheral inflammation as measured by an increase in activated CD8^+^ T cells [26, 29]. To examine the consequences of MRV infection in 5XFAD mice, we first investigated whether MRV infects the brains of neonatal 5XFAD mice after peripheral infection using intraperitoneal (i.p.) injection (Fig. 1a). We evaluated MRV DNA in the brains of 5XFAD mice compared to WT B6.SJL mice at 7 days post infection (dpi) and found similar levels in the cortex (Fig. 1b). Studies of herpesviruses have demonstrated that expression of late viral transcripts is correlated with viral replication [45]. We therefore utilized expression of the putative late viral transcript, ORF69, as a proxy of viral replication and found similar levels of expression in 5XFAD and WT mice (Fig. 1c). We then tested whether 5XFAD mice are susceptible to CD4^+^ thymocyte and peripheral CD4^+^ T cell depletion after neonatal MRV infection. Indeed, we determined that 5XFAD mice develop depletion of CD4 SP and DP thymocytes as well as CD4^+^ T cell depletion and an increase in activated CD8^+^ T cells in the periphery (Fig. 1d, e). Finally, since our data suggested active MRV replication in the brain, we evaluated expression of multiple inflammatory genes from the cortex of infected 5XFAD and WT mice compared to uninfected WT mice. We detected an increase in certain transcripts for microglia (*Iba1*), disease-associated microglia (*Trem2*), disease-associated astrocytes (*Gfap*), and proinflammatory cytokines (*Il6*, *Il-1b*) (Fig. 1f) [46, 47].. Together, these data demonstrate 5XFAD mice are equally susceptible to MRV infection as WT mice, with functional peripheral effects (T cell depletion) and viral replication in the brain inducing increased expression of inflammatory genes.

### Aβ interacts with MRV particles in vitro and disrupts infection in vivo

Studies proposing Aβ as an antimicrobial peptide are predicated on the findings that, like other antimicrobial peptides, Aβ interacts with pathogens to disrupt infection [2–4, 48]. Furthermore, Aβ has been demonstrated to interact with herpesvirus particles, including HHV-6, and inhibit HSV infection in vitro [3, 5]. We therefore evaluated Aβ interactions with MRV. We generated monomeric, oligomeric or fibrillar Aβ_42_ in a 10 μM suspension to differentiate which form of Aβ_42_ may interact with MRV particles from purified viral stocks. We incubated viral particles with Aβ_42_ for 2 h, then used negative staining transmission electron microscopy (NS-TEM) to identify nanogold-labeled Aβ (> 1.4 nm). We found that compared to PBS (Blank) and nanogold controls, all forms of Aβ_42_ interacted with viral particles (Fig. 2a). This was true for the majority of viral particles imaged, although the percent of interaction was lowest for monomeric Aβ_42_ (Fig. 2b). Interestingly, in general there were no large aggregates of Aβ_42_ associated with viral particles, nor was there direct interaction of Aβ_42_ fibrils with viral particles even though the monomers/oligomers in the suspension did interact (Fig. 2a). Our data does not differentiate if the nanogold-labeled Aβ interacting with MRV particles in the oligomeric and fibrillar Aβ_42_ preparations was oligomeric or monomeric. Although we did not observe MRV interactions with large Aβ_42_ aggregates as previously reported [5], these data demonstrate that MRV does interact with certain forms of Aβ_42_ in vitro.

We next evaluated whether Aβ_42_ has antimicrobial properties against MRV. Inhibitory properties of antimicrobial peptides, including Aβ_42_, were suggested to be associated with Aβ oligomerization, which is the preparation of Aβ_42_ we observed to have the highest percentage of interaction with MRV (Fig. 2b) [2, 5, 49–51]. We therefore tested the impact of oligomeric-Aβ_42_ on MRV infection. Purified viral stock was incubated with 10 μM purified oligomeric-Aβ_42_ for 2 h, then BALB/c mice were infected on postnatal day 0 (P0). We observed that compared to control infection, oligomeric-Aβ_42_ incubation with MRV resulted in a marked reduction in CD4 SP and DP depletion as well as a reduction in MRV DNA in the thymus (Fig. 2c, d). These findings confirm that Aβ_42_ interacts with MRV particles in vitro and then subsequently disrupts MRV infection in vivo.

### MRV infection does not increase cortical Aβ deposition of 5XFAD mice after peripheral infection

Human roseoloviruses infections typically occur in childhood and demonstrate high seroprevalence in adulthood [21]. The impact and incidence of reinfection and frequency of reactivation of roseoloviruses is unknown, including for MRV. We therefore took several different approaches to evaluate the influence of MRV infection on the development of Aβ load. Because human roseoloviruses infections typically occur early in life, we performed neonatal i.p. infection of P0 neonatal 5XFAD mice and assessed Aβ load at 6 months (Fig. S1a). While we observed that the percentage of peripheral CD4^+^ T cell per total T cells was decreased at 10 dpi (Fig. S1b) similar to what we have published previously in other mouse strains [26, 29], this effect was transient and no longer present at 6 months post infection (mpi) (Fig. S1c). Importantly, we did not observe a change in Aβ plaque load in the cortex 6 mpi after neonatal MRV infection (Fig. S1d, e). Since accumulation of tau can also be pathogenic, we evaluated immunostaining for total tau and phosphorylated tau (p-tau) levels in mock and MRV infected mice and did not observe increased levels of either after MRV infection (Fig. S1f-i). One possibility for the absence of an effect of MRV on Aβ plaque load could be explained by a saturation effect of Aβ by 6 months of age. We therefore tested whether neonatal MRV infection could accelerate the seeding of Aβ plaques in 6-week-old 5XFAD mice, a timepoint before the formation of extracellular Aβ plaques (Fig. S1j) [30]. After neonatal i.p. infection, we did not detect a difference in Aβ immunoreactivity in the cortex of mock- and MRV-infected mice (Fig. S1k, l). These findings suggest that neonatal MRV infection did not facilitate Aβ deposition when assessed at two timepoints (pre- and post-plaque).

Extracellular Aβ plaque formation begins at around 8 weeks in 5XFAD mice in the cortex [30]. It may be possible that pre-existing plaques in conjunction with viral infection are required to trigger downstream neuropathogenesis. We therefore also performed i.p. infections at 9 weeks, after the onset of plaque formation, and evaluated Aβ plaque load at 3 mpi, at 6 months of age, a timepoint characterized by high Aβ plaque burden (Fig. 3a). Although we previously observed CD4^+^ T cell depletion at 10 dpi, we did not observe prolonged CD4^+^ T cell depletion in the periphery or significant levels of MRV DNA in the cortex of 5XFAD mice at 3 mpi (Fig. 3b, c). Moreover, there was no change in cortical Aβ plaque pathology between mock- and MRV-infected mice when assessed using two methodological approaches, histology and enzyme-linked immunosorbent assay (ELISA) (Fig. 3d–i). Similarly, there was no difference in Iba1^+^ microglial coverage in the cortex and hippocampus of mock- and MRV-infected mice (Fig. 3j–l). These results suggest that adult MRV infection does not facilitate Aβ progression and formation in a mouse model of amyloidosis. Taken together, these data suggest that peripheral infection with MRV did not accelerate or increase Aβ formation in 5XFAD mice, despite an acute inflammatory response in the CNS and periphery.

### Adult CNS infection with MRV results in acute inflammation but does not increase Aβ load

One possible explanation for the discrepancy between our findings and previous work on the effects of viral pathogens on Aβ deposition could conceivably be differences in the route of infection [5]. One study that demonstrated enhanced Aβ aggregation in 6-week-old mice after HSV-1 infection was performed using direct, intracranial hippocampal infection [5]. Therefore, we performed a similar experiment in which we injected MRV (4 × 10^6^ genome copies of virus stock) bilaterally into the hippocampus and evaluated Aβ load 72 h post infection (hpi) (Fig. 4a). We used a semi-purified viral stock, which showed similar infection characteristics compared to unpurified stocks, to reduce any impact of non-viral components on Aβ load and inflammation (Fig. S2). We detected MRV DNA in the hippocampus of all infected mice (Fig. 4b). However, we detected no change in Aβ immunoreactivity in the hippocampus and cortex (Fig. 4c–e), or formation of extracellular Aβ plaques (Fig. 4f). Similar to neonatal, peripheral infections, MRV intracranial infection stimulated an acute, inflammatory response marked by an upregulation in microglial, astrocytic, and pro-inflammatory cytokine-related gene transcripts (Fig. 4g). These data were supported by an increase in Iba1^+^ microglial coverage in the hippocampus of MRV-infected brains (Fig. 4h, i). There was also an increase in total tau immunoreactivity in the cortex (Fig. S3a–c), which was potentially a response to focal inflammation [52–54]. There was not a statistically significant increase in total tau immunoreactivity in the hippocampus or phosphorylated tau in the cortex or hippocampus (Fig. S3e-g). Our findings suggest that MRV can induce inflammation in the brain after direct CNS infection, but neither MRV nor inflammation secondary to infection are sufficient to rapidly increase Aβ deposition in the brain.

### HHV-6 abundance is similar between human AD and control brains

**Fig. 5:**
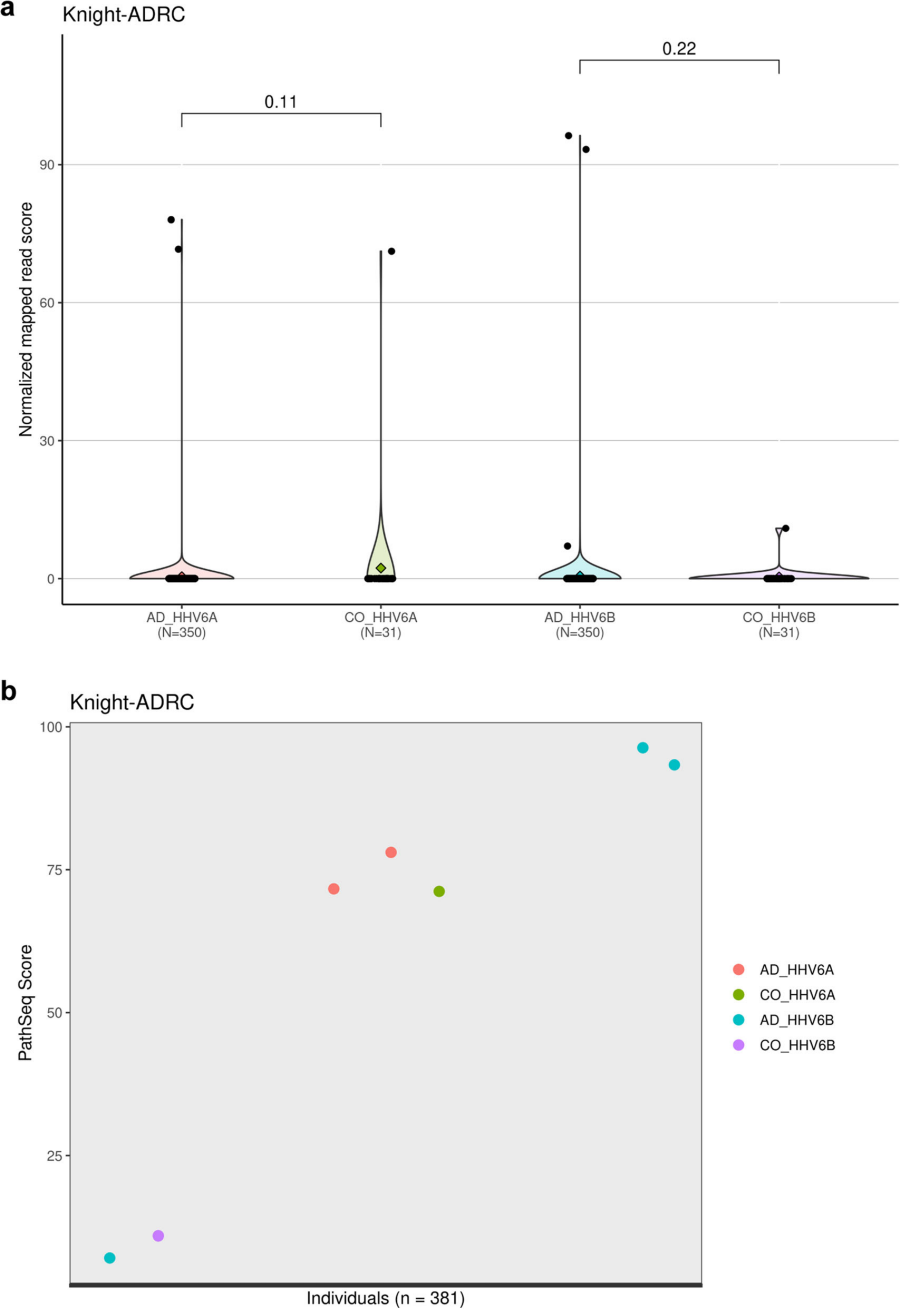
Relationship between viral abundance and AD status. **A** Comparisons of HHV-6A and -6B viral abundance between AD (*N* = 350) and non-demented controls (*N* = 31) in the Knight-ADRC cohort. Diamonds in violin plot: mean of group. Shape of violin plot: distribution of normalized PathSeq scores across each group. The *p*-values representing the significance of difference between the viral abundance in AD and controls groups for HHV-6A and -6B are shown at the top of corresponding violin plots. The *p*-values were obtained by performing a non-parametric Wilcoxon Rank Sum test in order to determine if there was any relationship between viral abundance and AD classification. Abbreviations: AD, Alzheimer Disease; CO, controls; HHV, human herpes virus. **B** Abundance of HHV-6A and HHV-6B in Knight-ADRC. HHV-6A and HHV-6B normalized PathSeq scores displayed in an increasing order for all the samples (AD and controls) in the Knight-ADRC cohort. Normalized PathSeq score based on the number of reads that align with a references taxon and indicates the amount of evidence that a taxon is present in a particular individual

RNAseq data from the Knight-ADRC cohort was available from frozen brain parietal cortex tissue for 381 individuals. Only a subset of the Knight-ADRC cohort was considered for this study where clinical data for each individual included Clinical Dementia Rating (CDR) score at the time of death and neuropathological assessment (Table 1). We performed RNA-seq analysis similar to previously described studies [36–39], aligning reads to the human genome followed by alignment of unmatched reads to 25,799 pathogens, including 118 human viruses [19]. Identification of HHV-6 transcripts was rare in non-AD control and AD samples (HHV-6A: 1/31 in non-AD controls and 2/350 in AD; HHV-6B: 1/31 in non-AD controls and 3/350 in AD) (Fig. 5a). HHV-7 transcripts were not identified in either the non-AD or AD samples. Upon arranging individuals based on their PathSeq scores, we observed no clear distinction of viral abundance between the individuals with AD vs. the controls (Fig. 5b). The normalized PathSeq score was based on the number of reads that align with a references taxon and indicates the amount of evidence that a taxon is present in a particular individual [44]. Moreover, upon checking the relationship between viral abundance and AD classification using the non-parametric Wilcoxon Rank Sum test, we observed no significant difference for either HHV-6A (*P*-value = 0.11) or HHV-6B (*P*-value = 0.22) between AD and controls groups (Fig. 5a). Therefore, our data, similar to a recent study [14], does not support an association between human roseolovirus infection and AD in the analysis of this cohort.

**Table 1:**
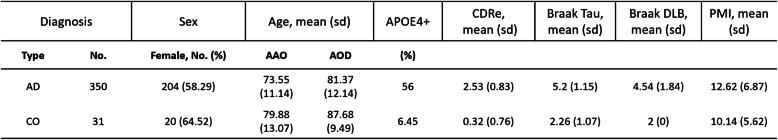
Demographics of RNAseq samples from Knight-ADRC cohort. Abbreviations: SD, standard deviation; APOE4+, apolipoprotein E4 carriers; CDRe, Clinical Dementia Rating at death; Braak DLB, Braak stages for dementia Lewy body; PMI, postmortem interval in hours; AD, Alzheimer’s disease; CO, controls; AAO, age at disease onset in years; AOD, Age of death in years

## Discussion

The relationship between the human roseoloviruses and AD has been of significant recent interest. While several studies have reported an association between HHV-6 or HHV-7 and AD [6, 17–19, 55], others have provided data that there is not a relationship [14, 20, 56, 57]. Currently, mouse models of human HHV-6 are limited. MRV is a natural murine pathogen and is highly related to HHV-6 and HHV-7, sharing genetic, morphologic and disease homology [26]. Moreover, our studies demonstrate that MRV enters the CNS and replicates in the cortex. Studying MRV therefore affords an opportunity to perform in vivo studies to model human roseolovirus neuropathogenesis, especially its relation to neuropathological features of AD-like disease in mice.

The antimicrobial protection hypothesis of Aβ posits that pathogens causing persistent or recurrent infections in the CNS are sequestered by Aβ, triggering a hyperreactive innate immune response that results in Aβ fibrillization and consequently contributes to AD [50]. Indeed, we found that purified Aβ_42_ interacted with MRV particles in vitro and, subsequent to this interaction, disrupted infection in vivo*.* We therefore evaluated Aβ deposition after MRV infection to test the antimicrobial hypothesis for MRV infection. Natural infection with MRV likely occurs through secretions [58], but i.p. infection would similarly require MRV to enter the CNS from the periphery, which we found did indeed occur after neonatal infection. However, in our studies, we did not observe an increase in Aβ after MRV infection of 5XFAD mice. It is important to note that, similar to other recent studies [5, 59], we focused on expression of human transgenic Aβ and did not evaluate endogenous mouse APP expression, which has been suggested to have antimicrobial properties [4]. Factors such as the impact of MRV infection on APP expression driven by the endogenous mouse promoter compared to the Thy-1 promoter remain to be explored. We approached investigating the impact of infection in multiple ways, including utilizing two routes of infection (peripheral, i.p. or direct CNS, intracranial), infecting at various timepoints (pre- or post-Aβ plaque development), as well as multi-timepoint assessments post-infection (acute or chronic). None of these scenarios resulted in increased Aβ load, suggesting that MRV does not facilitate the progression of Aβ accumulation over time.

Eimer et al. demonstrated that high dose intracranial HSV-1 infection in 5XFAD mice induced Aβ plaque formation by 72 h in 6-week-old-mice, with colocalization of Aβ plaques with HSV-1 viral particles [5]. A subsequent study that attempted to repeat these results with defined HSV-1 strains, albeit with potentially lower multiplicity of infection, did not observe colocalization of HSV-1 and Aβ plaques [60]. Neither study quantified the Aβ plaque load after infection, an important analysis given the rapid plaque formation reported in the former study. We therefore performed similar intracranial infections of 6-week-old 5XFAD mice with MRV and did not observe extracellular Aβ plaque formation. A caveat of these studies is intracranial injection was not performed at a later time point when Aβ plaques have begun to accumulate, which conceivably could be further seeded by a virus. Our studies were limited by the current lack of tools available to evaluate localization of MRV in tissues such as MRV-specific antibodies. Although we cannot compare doses between MRV and HSV-1, we cannot rule out that higher doses of MRV could induce Aβ formation.

The association between AD and human roseolovirus has been the focus of numerous multi-omics studies. A study of multiple cohorts utilizing genomic, transcriptomic and proteomic data reported an association between AD and human roseoloviruses, especially HHV-6A and HHV-7 [19]. Furthermore, the data suggested that certain pathways associated with inflammation and viral immune response were differentially expressed in AD patients compared to non-AD controls. Two subsequent studies have called these findings into question. One study performing a reanalysis of the data with different statistical methods showed no difference [20]. An additional study of three AD cohorts using RNAseq and digital droplet PCR analysis did not establish differences in human roseolovirus detection between AD and control samples [14, 20]. Similar to the study by Allnut, et al., we did not identify an association between human roseoloviruses and AD in our RNAseq analysis of the Knight-ADRC cohort. HHV-6A and HHV-6B were only detected in a few patients in both the AD and non-AD control samples. Moreover, given the small number of positive patients, we could not establish a statistically significant PathSeq score between the AD and non-AD groups. Although our data do not rule out a potential contribution of human roseoloviruses in the pathogenesis of AD, especially at an individual level, it does support recent data suggesting that roseoloviruses are not associated with AD at the population level.

Although herpesviruses have garnered interest as causative contributors to development of AD due to their neurotropism and chronic infections with periodic reactivation, utilizing -omics analysis from a single time point does not allow for evaluation of the viral load during, and frequency of reactivation. Moreover, selectively sampling CNS tissue at times of reactivation is not possible under most circumstances in human subjects. Several recent studies have suggested that treatment with antivirals for herpesvirus infections was associated with a decreased risk of dementia later in life compared to untreated patients [61–64]. In these studies, treatment generally was given in relatively short intervals considering the lifelong nature of herpesvirus infections, and many patients were treated with acyclovir, which has low efficacy against HHV-6 [65–67]. Prospective randomized control trials will likely address the use of herpes-specific antivirals for AD, although studying the impact of antiviral on reactivation in mice could provide a means to test efficacy in vivo*.* Indeed, a recent study using thermal stress to serially reactivate HSV-1 in mice demonstrated that intermittent stress resulted in HSV-1 reactivation, inflammation, and increased Aβ deposition in the brain [59]. While MRV reactivation has not been examined in detail, it is possible that MRV also requires induced reactivation through intermittent stress to promote Aβ accumulation. Additionally, reinfection with roseoloviruses in general is not well understood, including in the context of AD. Further evaluation of reinfection could provide important information regarding the role of roseoloviruses in AD. An important consideration for our studies and future studies is that the mechanism of MRV spread within tissue could impact Aβ accumulation. Human roseolovirus infection is thought to be largely cell-associated or through exosomes, which could make them less accessible to extracellular Aβ aggregation [68]. Perhaps higher viral loads resulting in increased extracellular MRV-Aβ interaction is necessary to increase Aβ plaque formation, which may be why we did not observe Aβ accumulation in 5XFAD mice that have an intact immune system.

While MRV infection did not result in increased Aβ, we determined that MRV interacts with Aβ, and that it infects the brain and acutely induces neuroinflammation. Whether MRV has cell-specific tropism is also unknown. It is possible that Aβ entrapment of viral pathogens facilitated Aβ seeding, but upregulation of disease-associated microglia rapidly assisted in the phagocytosis and removal of Aβ plaques before further seeding and spreading of Aβ could occur. However, as there were no differences between viral load in the cortex of WT and 5XFAD mice, if Aβ indeed sequestered MRV, it likely did not neutralize sufficient viral load to prevent further replication. Additional research could provide insight into CNS cell types that are especially vulnerable to infection which may result in Aβ-independent toxic effects. Furthermore, it has been suggested that genetic variation resulting in an altered immune response to pathogens could contribute to viral load and reactivation in AD [19, 69]. Our study utilized inbred mouse strains (5XFAD mice on a B6SJL background) that do not have immunodeficiency, but it is possible that MRV infection of mice with defects in genes involved in viral immune response could cause increased Aβ load due to higher viral burden during acute infection or reactivation. For example, miR-155 knockout mice crossed to an APP/PS1 mouse developed increased Aβ load in the cortex compared to APP/PS1 controls [19]. miR-155 has been suggested to be important in controlling herpesvirus latency and is downregulated during lytic HHV-6 replication in NK cells [70, 71]. MRV infection of miR-155 knockout, or other mice with altered viral immune response mice could provide new insight into the relationship between immune control of roseolovirus lytic and latent infection and AD.

Our findings in this study do not support or refute the antimicrobial protection hypothesis of Aβ. However, our results suggest that HHV-6 is not a main contributor for increased AD risk in humans at a population level. MRV infections, like human roseolovirus infections, are well controlled in immunocompetent hosts and do not result in mortality. Neonatal infection of WT and 5XFAD mice resulted in similar levels of MRV DNA and RNA in the cortex 7 dpi, suggesting Aβ has minimal, if any, antiviral properties towards neutralizing MRV after infection in 5XFAD mice. Although some markers of inflammation trended higher in the 5XFAD mice compared to WT littermates, these differences were not statistically significant. Despite this, a major finding in the current study was that MRV actively infects the brain and stimulates an inflammatory response, even after peripheral infection. Moreover, we found that Aβ interacts with MRV in vitro and, subsequent to this interaction, inhibits in vivo infection. In addition to further studies of the relationship between MRV and AD-like phenotypes in mice, MRV could be studied in other diseases characterized by CNS inflammation. Human roseoloviruses have been associated with several other inflammatory and autoimmune CNS diseases, including viral encephalitis, multiple sclerosis, and Rasmussen encephalitis [72–74]. Given the limitations of current mouse models of roseolovirus infection, MRV could be highly useful for performing deliberate, controlled studies to uncover the mechanistic role of a roseolovirus in CNS disease.

## Conclusion

Human roseoloviruses, HHV-6 and HHV-7 have recently been investigated for their role in AD, although studies in humans have provided controversial results. MRV is highly related to the human roseoloviruses, allowing for evaluation of the role of a natural murine roseolovirus in neuroinflammation and Aβ accumulation. Although Aβ interacted with MRV and disrupted infection, we found that MRV did not induce increased Aβ load after peripheral or direct CNS infection. However, we demonstrated that MRV actively infects the brain and induces neuroinflammation, providing a suitable in vivo system for further studies of the impact of roseolovirus infection of the brain.

## Supplementary Information

**Additional file 1: Supplementary Fig. 1.** Peripheral, neonatal MRV infection in 5XFAD mice does not accelerate Aβ plaque burden. **A,** Schematic of experimental paradigm (6 mpi). **B, C**, Percent of CD4^+^ and CD8^+^ T cells in plasma 10 dpi (**B**) or 6 mpi (**C**). **D, E**, HJ3.4B^+^ immunostaining for Aβ plaque load in the cortex. **F–I**, Staining and quantification for Tau5^+^ total tau (**F, G**) and AT8^+^ phosphorylated tau in the cortex (**H, I**). **J**, Schematic of experimental paradigm (6 wpi). **K, L**, Percent area HJ3.4B^+^ Aβ immunoreactivity (Aβ - IR) in cortex. Dpi: days post infection. Mpi: months post infection. Wpi: weeks post infection. p-tau: phosphorylated tau. Scale bar: 500 μm. Data expressed as mean ± SEM, student’s t-test (**B, C, E, G, J, M**). No statistical comparisons are significant unless indicated.

**Additional file 2: Supplementary Fig. 2.** Comparisons between MRV purified and unpurified stocks**. A – C,** Stocks were created from in vivo passage and collection of 7 day post neonatal infection thymi and were used directly (MRV Stock) or semi-purified (MRV Purified). BALB/c mice were mock- or MRV-infected with MRV Stock or MRV Purified via i.p. injection on P0 then were evaluated by flow cytometry for percent CD4^+^ cells per total CD3^+^ cells from the spleen at 7dpi (**A**) or weight at 7dpi (**B**). **C,** Copies of MRV DNA per mL of stock were evaluated by qPCR.

**Additional file 3: Supplementary Fig. 3.** Effects of acute, intrahippocampal MRV infection on tau pathology. **A**, Schematic of experimental paradigm. **B – G,** Staining and quantification for Tau5^+^ total tau (**B, C, D**) and AT8^+^ phosphorylated tau in the cortex (**E, F, G**). p-tau: phosphorylated tau. Scale bar: 500 μm. Data expressed as mean ± SD, student’s t-test. *P < 0.05. ns = not statistically significant. No statistical comparisons are significant unless indicated.

## Data Availability

Generated datasets used for analyses in this study are available from the corresponding author upon reasonable request.

## Abbreviations

5XFAD: 5 familial AD mutations
Aβ: amyloid-β
AD: Alzheimer disease
APP: amyloid precursor protein
CNS: central nervous system
CDR: Clinical Dementia Rating
DNA: Deoxyribonucleic acid
DP: double positive
Dpi: days post infection
ELISA: Enzyme-linked immunosorbent assay
Gfap: Glial fibrillary acidic protein
HCl: Hydrochloric
HHV: human herpesvirus
Hpi: hours post infection
HRP: Horseradish peroxidase
HSV: herpes simplex virus
Iba1: Ionized calcium binding adaptor molecule 1
IL-6: Interleukin-6
IL-1β: Interleukin-1 beta
i.p.: intraperitoneal
Knight ADRC: Knight Alzheimer’s Disease Research Center
miR: microRNA
Mpi: months post infection
MRV: murine roseolovirus
NS-TEM: Negative staining transmission electron microscope
PSEN1: Presenilin 1
RNAseq: Ribonucleic acid sequencing
qPCR: Quantitative polymerase chain reaction
SEM: Standard error of the mean
SP: single positive
TBS: Tris-buffered saline
Trem2: Triggering Receptor Expressed On Myeloid Cells 2
Wpi: weeks post infection
WT: wild-type
