# Supplementary figures and images for: Murine roseolovirus does not accelerate amyloid-β pathology and human roseoloviruses are not over-represented in Alzheimer disease brains
Source: Mol Neurodegener. 2022 Jan 15;17:10. doi: 10.1186/s13024-021-00514-8 (PMC8760754; doi:10.1186/s13024-021-00514-8)

Figure S1

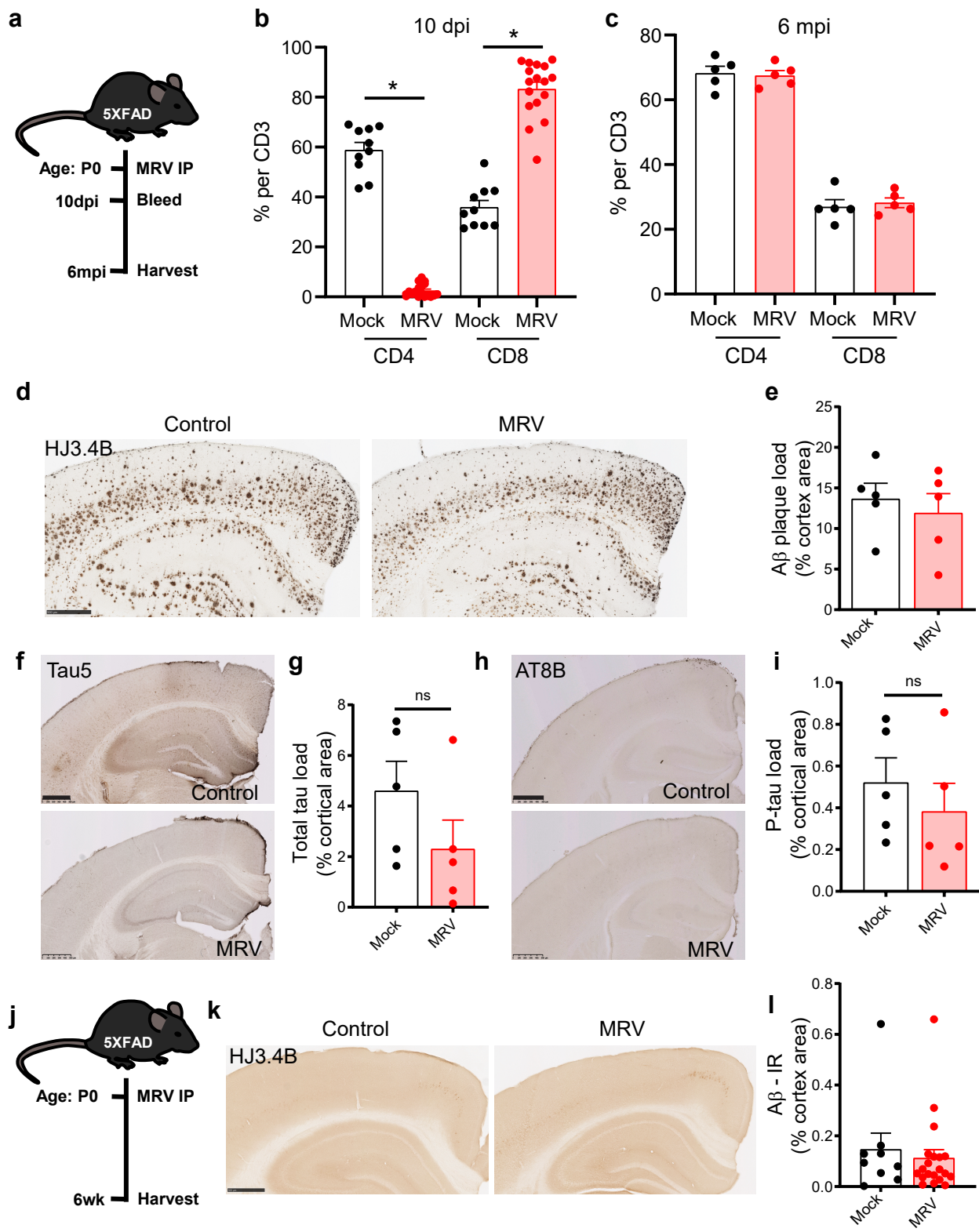

Supplement: Supplementary file 1 — Additional file 1: Supplementary Fig. 1. Peripheral, neonatal MRV infection in 5XFAD mice does not accelerate Aβ plaque burden. A, Schematic of experimental paradigm (6 mpi). B, C, Percent of CD4+ and CD8+ T cells in plasma 10 dpi (B) or 6 mpi (C). D, E, HJ3.4B+ immunostaining for Aβ plaque load in the cortex. F–I, Staining and quantification for Tau5+ total tau (F, G) and AT8+ phosphorylated tau in the cortex (H, I). J, Schematic of experimental paradigm (6 wpi). K, L, Percent area HJ3.4B+ Aβ immunoreactivity (Aβ - IR) in cortex. Dpi: days post infection. Mpi: months post infection. Wpi: weeks post infection. p-tau: phosphorylated tau. Scale bar: 500 μm. Data expressed as mean ± SEM, student’s t-test (B, C, E, G, J, M). No statistical comparisons are significant unless indicated. [file 13024_2021_514_MOESM1_ESM.pdf]

# Figure S2

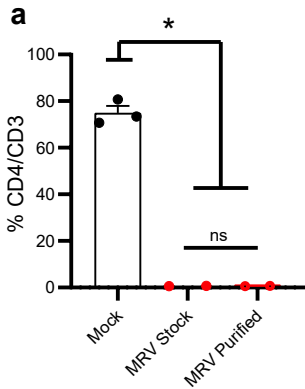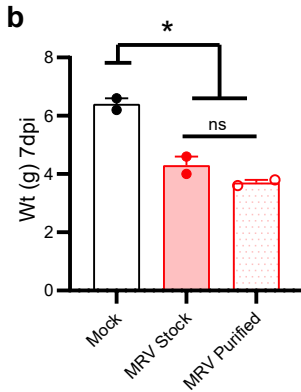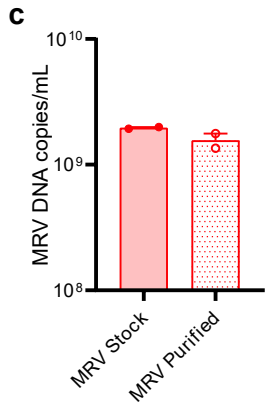

Supplement: Supplementary file 2 — Additional file 2: Supplementary Fig. 2. Comparisons between MRV purified and unpurified stocks. A – C, Stocks were created from in vivo passage and collection of 7 day post neonatal infection thymi and were used directly (MRV Stock) or semi-purified (MRV Purified). BALB/c mice were mock- or MRV-infected with MRV Stock or MRV Purified via i.p. injection on P0 then were evaluated by flow cytometry for percent CD4+ cells per total CD3+ cells from the spleen at 7dpi (A) or weight at 7dpi (B). C, Copies of MRV DNA per mL of stock were evaluated by qPCR. [file 13024_2021_514_MOESM2_ESM.pdf]

Figure S3

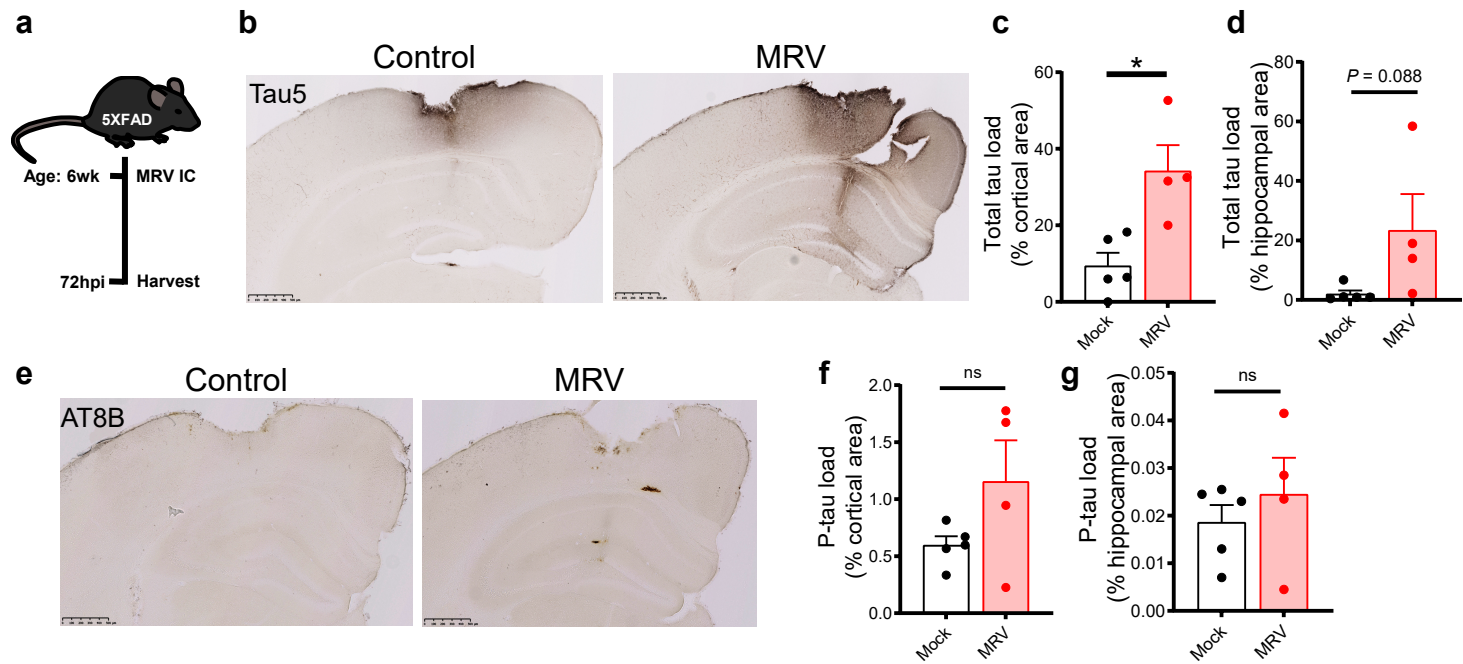

Supplement: Supplementary file 3 — Additional file 3: Supplementary Fig. 3. Effects of acute, intrahippocampal MRV infection on tau pathology. A, Schematic of experimental paradigm. B – G, Staining and quantification for Tau5+ total tau (B, C, D) and AT8+ phosphorylated tau in the cortex (E, F, G). p-tau: phosphorylated tau. Scale bar: 500 μm. Data expressed as mean ± SD, student’s t-test. *P < 0.05. ns = not statistically significant. No statistical comparisons are significant unless indicated. [file 13024_2021_514_MOESM3_ESM.pdf]
